# The Click-On gamma probe, a second-generation tethered robotic gamma probe that improves dexterity and surgical decision-making

**DOI:** 10.1007/s00259-021-05387-z

**Published:** 2021-05-25

**Authors:** Samaneh Azargoshasb, Simon van Alphen, Leon J. Slof, Giuseppe Rosiello, Stefano Puliatti, Sven I. van Leeuwen, Krijn M. Houwing, Michael Boonekamp, Jeroen Verhart, Paolo Dell’Oglio, Jos van der Hage, Matthias N. van Oosterom, Fijs W. B. van Leeuwen

**Affiliations:** 1 Interventional Molecular Imaging-Laboratory, Department of Radiology, Leiden University Medical Center, Leiden, the Netherlands; 2 Department of Urology, Netherlands Cancer Institute-Antoni van Leeuwenhoek Hospital, Amsterdam, the Netherlands; 3 Instrumentele zaken ontwikkeling, facilitair bedrijf, Leiden University Medical Center, Leiden, the Netherlands; 4 Department of Urology and Division of Experimental Oncology, Urological Research Institute IRCCS San Raffaele Scientific Institute, Milan, Italy; 5 Department of Urology, University of Modena and Reggio Emilia, Via del Pozzo, 71, 41124 Modena, Italy; 6 ORSI Academy, Melle, Belgium; 7 Department of Urology, Onze Lieve Vrouw Hospital, Aalst, Belgium; 8 Department of Urology, ASST Grande Ospedale Metropolitano Niguarda, Milan, Italy; 9 Department of Surgery, Leiden University Medical Center, Leiden, the Netherlands

**Keywords:** Radioguided surgery, Robotic surgery, Surgical training, Precision surgery, Performance assessment, Image-guided surgery

## Abstract

**Purpose:**

Decision-making and dexterity, features that become increasingly relevant in (robot-assisted) minimally invasive surgery, are considered key components in improving the surgical accuracy. Recently, DROP-IN gamma probes were introduced to facilitate radioguided robotic surgery. We now studied if robotic DROP-IN radioguidance can be further improved using tethered Click-On designs that integrate gamma detection onto the robotic instruments themselves.

**Methods:**

Using computer-assisted drawing software, 3D printing and precision machining, we created a Click-On probe containing two press-fit connections and an additional grasping moiety for a ProGrasp instrument combined with fiducials that could be video tracked using the Firefly laparoscope. Using a dexterity phantom, the duration of the specific tasks and the path traveled could be compared between use of the Click-On or DROP-IN probe. To study the impact on surgical decision-making, we performed a blinded study, in porcine models, wherein surgeons had to identify a hidden ^57^Co-source using either palpation or Click-On radioguidance.

**Results:**

When assembled onto a ProGrasp instrument, while preserving grasping function and rotational freedom, the fully functional prototype could be inserted through a 12-mm trocar. In dexterity assessments, the Click-On provided a 40% reduction in movements compared to the DROP-IN, which converted into a reduction in time, path length, and increase in straightness index. Radioguidance also improved decision-making; task-completion rate increased by 60%, procedural time was reduced, and movements became more focused.

**Conclusion:**

The Click-On gamma probe provides a step toward full integration of radioguidance in minimal invasive surgery. The value of this concept was underlined by its impact on surgical dexterity and decision-making.

## Introduction

The effectiveness of surgery has been posed as a balance between decision-making and dexterity [1]. Image-guided surgery technologies are generally intended to improve the first. For example, in the last decades, the availability of dedicated decision-making tools, e.g., radiopharmaceuticals such as ^99m^Tc-radiocolloids [2], ^99m^Tc-PSMA I&S [3, 4], and [^99m^Tc-EDDA/HYNIC]-octreotate [5] or dedicated gamma probe modalities [6], has enhanced the cognitive ability of surgeons, allowing them to make critical decisions during complex oncological resections. However, it is not always clear if and how such image-guided decision-making impacts on the completion of surgical tasks. Especially in highly technical minimally invasive procedures such as (robot-assisted) laparoscopic surgery, it can be challenging to make such assessments.

Where initially rigid laparoscopic gamma probes have been applied to support radioguidance in a minimally invasive laparoscopic setting [7], unique features related to robotic surgery (e.g., the precision and rotational freedom of the surgical instruments, as well as the master–slave concept whereby the surgeon manipulates the robotic instruments from a remote console) created a demand for a tethered gamma-probe concept. Robot-assisted sentinel lymph node procedures [8] and PSMA-guided salvage resection of lymph nodes [9] have helped confirm that the tethered (DROP-IN) gamma-probe concept allows the detector to match the robotic instruments’ degrees of freedom and facilitates autonomous control by the operating surgeon [6]. In fact, the DROP-IN technology was shown to improve the nodal detection rates in relation to the rigid laparoscopic gamma probe [10]. Building on these pioneering efforts, there are now two companies that have started to commercialize the DROP-IN technology: Sensei (Lightpoint Medical Ltd.; single use) and DROP-IN CXS-OP-DP (Crystal Photonics GmbH; sterilizable).

The lack of oncological outcome data makes most clinical image-guided surgery studies descriptive and suggestive at best. Exceptions to the rule are represented by reports for 5-ALA (*N* = 322) [11], sentinel node procedures in melanoma patients (*N* = 2001) [12], and ^99m^Tc-PSMA (*N* = 121) [13]. Nonetheless, clinical studies that describe image-guided surgery techniques remain very popular among both researchers and surgeons using molecular imaging. Interestingly, our experience with indocyanine green (ICG)-^99m^Tc-nanocolloid (*N* > 1500) indicates that surgeons that have used this form of hybrid image guidance no longer want to proceed without the real-time high-resolution images, despite clear improvement in oncological outcome data [14–16]. Similarly, due to the abovementioned improvements, robotic surgeons that have used the DROP-IN gamma probe [9, 10] no longer wish to convert back to using traditional laparoscopic gamma probes. These examples underscore that imaging guidance technologies apparently also just provide value by aiding decision-making and perhaps by enhancing surgical dexterity. Identification of the performance metrics that impact these features could thus help support the value determination of (future) image-guided surgery technologies.

In this frame, motion analysis is considered as an objective tool to study behavior, e.g., animal migration through the desert [17], analysis of cognitive impairment in patients [18], cell movement [19], and surgical performance [20]. Advanced 3D virtual instrument tracking is already used to provide quantitative performance assessment to assess tasks that were performed with the da Vinci robotic training console [21, 22]. The same concepts, coupled with mechanical instrument tracking, have been put forward as a tool to discriminate novice from expert surgical ability [23]. While showing clear potential, access to the latter technology is very restricted, limiting exploitation during the evaluation of new image guidance modalities and also limiting future expansion to coming robotic platforms (e.g., Versius, Revo-I, Senhance). In this sense, alternative, video-based instrument tracking strategies [24–26] provide a more generic means to map the *x*, *y*, *z*-paths along which surgical instruments move during minimal invasive procedures.

Since the creation of new image-guided surgery procedures is as much about engineering as it is about analyzing the technologies’ impact on performance, the aim of the current study was to (1) develop a second-generation—easy to use—tethered Click-On gamma probe that is integrated on top of the surgical instrument and provides surgical guidance while preserving instrument utility, (2) use marker-based video tracking to map the *x*, *y*, *z*-path of surgical instruments to assess how the new Click-On probe enhances dexterity relative to the DROP-IN technology, and (3) use computer-assisted movement analysis to quantify if and how Click-On probe enhances decision-making during porcine surgery.

## Methods

### Click-On probe construction

**Fig. 1:**
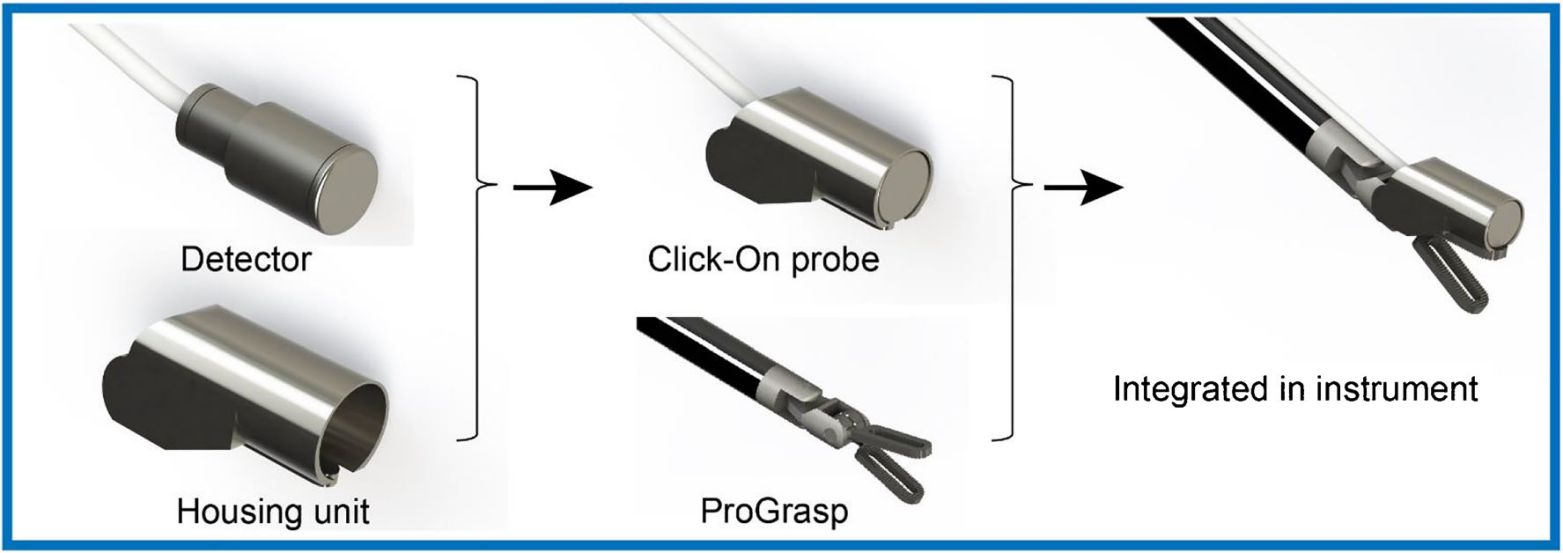
Schematic overview of the Click-On gamma probe design. Attaching the Click-On gamma probe to a ProGrasp instrument essentially yields a “smart” instrument that facilitates gamma-ray detection, while at the same time preserving its grasping function

The construction of the prototype Click-On gamma probe is depicted in Fig. 1. A scintillator (GAGG(Ce); resistant to high temperatures and compatible with 25–600 keV gamma rays) and photodiode are integrated in a tungsten (G17B) collimator surrounded by a unique housing mount. This housing was first tested and fabricated via 3D printing using Tough2000 material in a Formlabs 3B printer (Formlabs, Somerville, USA). This housing not only tightly holds the detector but it also allows for the detector to be connected to the ProGrasp forceps robotic instrument (Intuitive Surgical, Sunnyvale, CA, USA) using two attachment points (a press fit in the opening of the jaw and clamping behind the joint axis along which the jaw rotates). It should be noted that the back end of the housing contained an additional gripping area to also facilitate grasping by the ProGrasp instrument, when the detector is not clicked on the instrument. The fully assembled “smart-instrument” could be inserted through a 12-mm trocar and connected to a Crystal Probe—automatic (SG04) read-out module (Crystal Photonics, Berlin, Germany) using amplification electronics. This provided both an audible and numerical feedback/read-out when radioactivity (e.g., radiopharmaceutical) was detected with the Click-On probe.

For sentinel lymph node procedures, it has been reported that the activity in the nodes is typically 1% of the injected activity [6, 27]. To investigate the Click-On probe’s ability to identify a “low” activity object (i.e., 1 MBq ^99m^Tc source) in the vicinity of “high” activity background signals (i.e., 100 MBq ^99m^Tc source), a phantom experiment was performed. In this experiment, the distance between the sources was varied in the range of 0–5 cm (steps of 1 mm). In this setting, three different operators determined the distance at which the low activity source was distinguishable.

### Instrument tracking

A vision-based instrument tracking system was built to help determine the position of the ProGrasp instrument tip in three dimensions (3D). To this end, a cyan-colored and rectangle-shaped marker was placed on one side of the ProGrasp. Since the Click-On probe was integrated on the ProGrasp tool, a different marker was required to track the other side: a three-ring yellow marker pattern, similar to what was described previously [24], was incorporated in the Click-On housing unit. Since here we do not have 360° rings around the whole instrument, additional small rectangle-shaped cyan-colored markers were placed on the sides of the instrument to achieve a smoother transition between the different tracking markers when the instrument is rotated along the roll axis. Custom computer-vision software was created to segment the markers (based on shape and color) in the endoscopic-video output. After checkerboard calibrations of the endoscope’s intrinsic and extrinsic camera parameters [28], it became possible to use the known geometry of the markers to determine the location of the ProGrasp instrument tip along the *x*, *y*, and *z*-axis. To determine the accuracy of this tracking method, we assessed the individual accuracies for the *x*, *y*, and *z* components. This was done at a typical working distance from the laparoscope in robotic surgery (i.e., 15 cm) within a volume of 10 × 10 × 10 cm^3^. Accuracy in this phantom set-up was defined as the deviation between the true distance and the calculated tracking distance as determined from eight independent measurements per component.

### Phantom exercise: DROP-IN versus click-on gamma probe

**Fig. 2:**
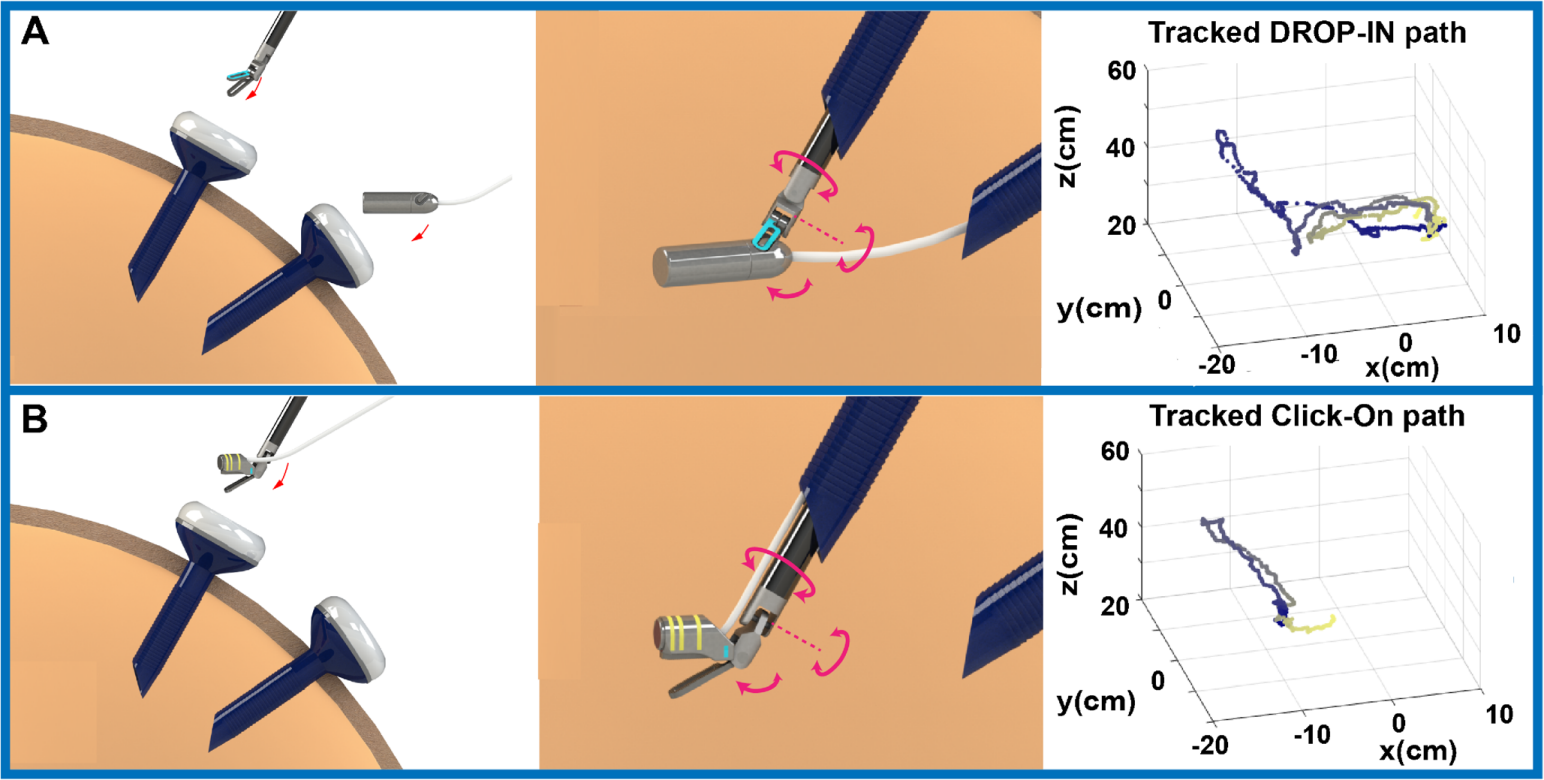
Schematic overview of the Click-On gamma probe vs. the DROP-IN gamma probe. **a** The DROP-IN gamma probe is inserted into the abdominal cavity through the assistant trocar (12 mm) then the surgeon picks it up with the surgical instrument, allowing for 6 degrees of freedom. **b** The Click-On gamma probe mounted on the ProGrasp instrument is inserted together with the instrument through a standard 12-mm trocar preserving the 6 degrees of freedom of maneuverability and leaving the assistant trocar completely free. The 3D trajectories are reconstructed for probe movements using the marker-based vision tracking system

To evaluate the Click-On performance compared to the DROP-IN gamma probe, we created a phantom setup that was designed to simulate the basic task of target identification, grasping, and removal. The phantom consisted of a 10-mm-thick polycarbonate plate with a recess for the DROP-IN and a radioactive source (^57^Co 0.2 MBq Disk; Eckert & Ziegler Isotope products), three target objects placed on top of the source, and a bucket for target collection, all located at defined locations. These locations were engineered such that each movement toward the source location or from source to collection buckets resulted in a specific peak in the *x*, *y*, or *z*-direction of the traveled path relative to the endoscopic view. Tests were performed by three participants using a da Vinci X robot (Intuitive Inc.) (Fig. 2).

In this exercise, the following ten movements were defined: (1) probe pick-up by the instrument at a predefined starting position (home), (2) probe movement from home toward the target, (3) target identification, (4) probe movement back to home, (5) dropping probe at home by instrument, (6) movement instrument to target, (7) pick-up target by instrument, (8) movement target to the collection bucket, (9) dropping target at collection bucket by instrument, and (10) move instrument back to home.

### In vivo exercise: radioguidance versus palpation

To investigate the feasibility of the designed Click-On gamma tracing in a true surgical environment, a porcine animal model underwent robot-assisted laparoscopic surgery using a da Vinci Xi robotic system. A radioactive source (^57^Co 0.2 MBq disk; Eckert & Ziegler Isotope products) was placed in the abdominal wall as a “hidden” surgical target. Subsequently, five similarly experienced robotic-urology fellows (i.e., all trained and experienced in robotic surgery, but without prior experience in radioguidance) were asked to sequentially identify the target’s location within a timeframe of 40 s. In their first attempt, the surgeons were only allowed to rely on palpation of the surgical view using the robotic instruments (blinded for the radioactive readout). In a second attempt, they were allowed to also use the radioguidance provided by the Click-On probe. Failure was considered when the lesion was not identified within 40 s. The clock was stopped when the target was identified.

### Analysis of the path traveled by the instruments

After preprocessing and filtering the data, the instrument’s 3D path over time could be reconstructed and analyzed. The data preprocessing consisted of indicating movements out of the detection range of the laparoscope, filling the missing track segments with a linear interpolation and application of a median filter to remove noise in the total tracking data. After reconstructing the movement trajectory, the features commonly used for movement analysis were calculated [23, 29, 30]. The total path length, completion time, angular dispersion, and straightness index were calculated for each trial, and for the features such as speed, acceleration, curvature, and smoothness, there is a number for each frame, and the median value is considered for the trial. Statistical significance was investigated using a paired-samples *t* test with the SPSS statistical software package (IBM SPSS Statistics for Windows, version 25.0).

In the in vivo experiment, to analyze the focus of the searching process, the percentage of time spent in each cubic centimeter of the 3D working volume (%s/cm^3^) was calculated and used to calculate a 3D color-coded density plot. For better interpretation of the data, the same concept was visualized in 2D (%s/cm^2^) using an overlay of the density plots on the surgical view. In addition, having the movement features from directionality analysis, a machine learning algorithm was used to determine the surgical dexterity based on movement trajectories, time, and final instrument positions. To this end, a logistic regression algorithm was used for binary classification for success or failure [23], after which the results from the sigmoid function were used as a score for each trail.

## Results

### Click-On gamma probe prototype

Through our engineering efforts, we were able to create a fully functional Click-On gamma probe prototype that could be attached to the ProGrasp instrument in two places. The final assembly could readily be inserted through a standard 12-mm trocar, while at the same time preserving the rotational freedom of the instrument and its tissue-grasping function. As the gamma detector essentially extends the ProGrasp instrument, ease of use for an experienced robotic surgeon is high.

By connecting the Click-On probe along the instrument axis, we slightly reduced the effective scanning range around the longitudinal axis of the instrument (−110 to +110°) relative to the original DROP-IN designs, which are grasped at a 45^o^ angle (−140 to +140°). Although the Click-On probe is primarily designed for use as attachment to the wristed surgical instruments, by including an additional grasping moiety at the proximal end of the housing, the Click-On probe could also function as a tethered probe. In this fashion, if needed, an extended scanning range of −140 to +140° can be facilitated around the longitudinal axis of the instrument.

Probe performance studies showed that the Click-On probe was able to distinguish a low activity from a high activity source (in a 1:100 ratio) when the distance between the two sources was 15 mm or higher. The marker-based tracking accuracy proved to be 1.10 ± 0.74 mm, 0.50 ± 0.53 mm, and 0.88 ± 0.99 mm in the *x*, *y*, and *z* directions, respectively.

### Dexterity improvements Click-On versus DROP-IN

**Fig. 3:**
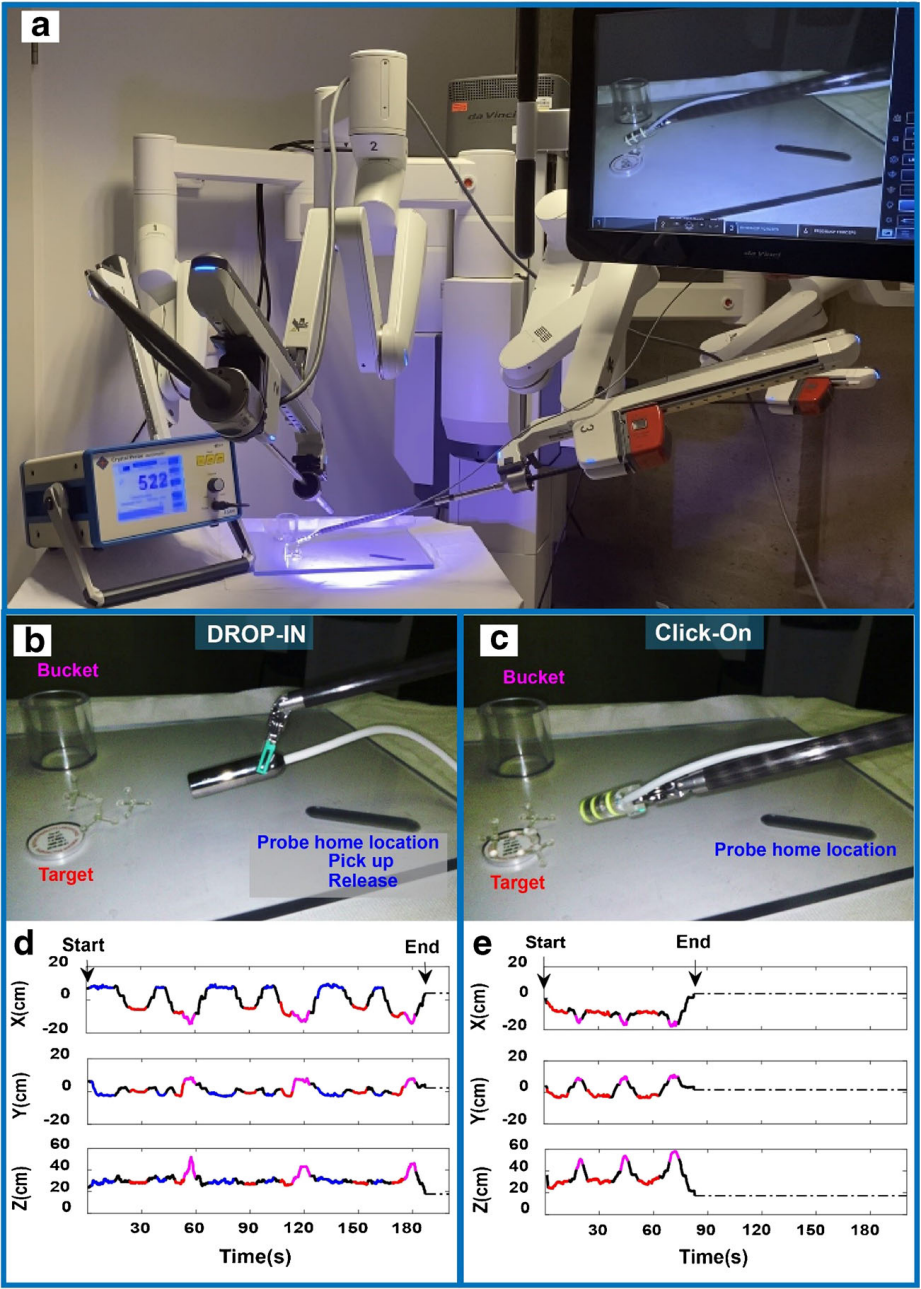
Phantom evaluation of Click-On vs. DROP-IN probe. **a** Overview of the robot-assisted phantom setting. **b**, **c** DROP-IN and Click-On probe in action on the Phantom Plate. **d**, **e** Color-coded diagram of the movement trajectory with DROP-IN and Click-On gamma probe (red—radioactive source location, purple—collection bucket, blue—the DROP-IN probe home location in the plate for releasing and picking up, and black—transition between locations). The coordinate system is such that *x*- and *y*-axes show the image plane with the center of the image as the origin and *z*-axis shows the distance from the camera

Using our custom target identification, grasping, and removal phantom with three targets, we have been able to classify the movement trajectories during an exercise performed using either the DROP-IN or the Click-On probe (Fig. 3). The specific locations of the detector placement, targets, and bucket (Fig. 3b, c) meant that specific movements converted to an identifiable pattern in the path trajectory. In fact, use of the Click-On probe reduced the number of movements required to perform the exercise by 40%. The directional metrics were color coded in the resulting *x*, *y*, and *z*-paths that were traveled during the exercise and specific movements therein (Fig. 3d, e). The characteristics of the movements such as distance traveled, speed, acceleration, curvature, smoothness, angular dispersion, and straightness index are extracted and analyzed, having the trajectory of the movement. One evident outcome over comparing the paths traveled using the two different gamma probes is that the path length with the Click-On probe (3.07 ± 0.40 m) was one third of that required with the DROP-IN probe (9.22 ± 1.82 m; *p* = 0.03). This also increased the straightness index with Click-On probe (0.037 ± 0.019 vs. 0.012 ± 0.009; *p* = 0.05). However, angular dispersion, smoothness, and path curvature were similar for both probes.

In addition to directional path analysis, the duration of the exercise also allowed us to incorporate time as factor. While the speed and acceleration of tool movement was identical in both procedures (see Table 1), the time spend on detecting and grasping of the target differed between the procedures. This meant that, in line with the abovementioned reduction of the path traveled, use of the Click-On probe reduced the procedural time three times (79.37 ± 11.70 s vs. 242.71 ± 62.15 s; *p* = 0.04).

**Table 1:** Phantom experiment results (mean and SD values)

	Time (s)		Path length (10^3^ mm)	Speed (mm/s)	Acceleration (mm/s^2^)	Straightness index	Angular dispersion	Smoothness 10^4^ mm/s^3^	Curvature 1/mm
DROP-IN	242.71 (62.15)		9.22 (1.82)	17.8 (4.36)	352.22 (144.14)	0.012 (0.009)	0.76 (0.03)	1.15 (0.52)	0.33 (0.07)
Click-On	79.37 (11.70)		3.07 (0.40)	23.67 (4.15)	529.43 (106.42)	0.037 (0.019)	0.78 (0.01)	1.43 (0.10)	0.39 (0.06)
Paired *t* test: *p* value	0.04		0.03	0.11	0.12	0.05	0.41	0.41	0.15
In vivo experiment results (mean and SD values)									
	Completion rate (%)	Time (s)	Path length (10^3^ mm)	Speed (mm/s)	Acceleration (mm/s^2^)	Straightness index	Angular dispersion	Smoothness 10^4^ mm/s^3^	Curvature 1/mm
Palpation only	20	34.00 (13.41)	1.81 (0.59)	29.42 (10.05)	703.80 (163.30)	0.035 (0.022)	0.79 (0.06)	1.72 (0.34)	0.35 (0.16)
Click-On radioguidance	80	27.20 (14.46)	1.67 (0.46)	25.75 (11.89)	649.13 (191.44)	0.019 (0.005)	0.74 (0.09)	1.68 (0.22)	0.32(0.12)
Paired *t* test: *p* value	0.057	0.07	0.60	0.61	0.52	0.15	0.32	0.82	0.77

### Impact of the Click-On gamma probe on surgical decision-making

**Fig. 4:**
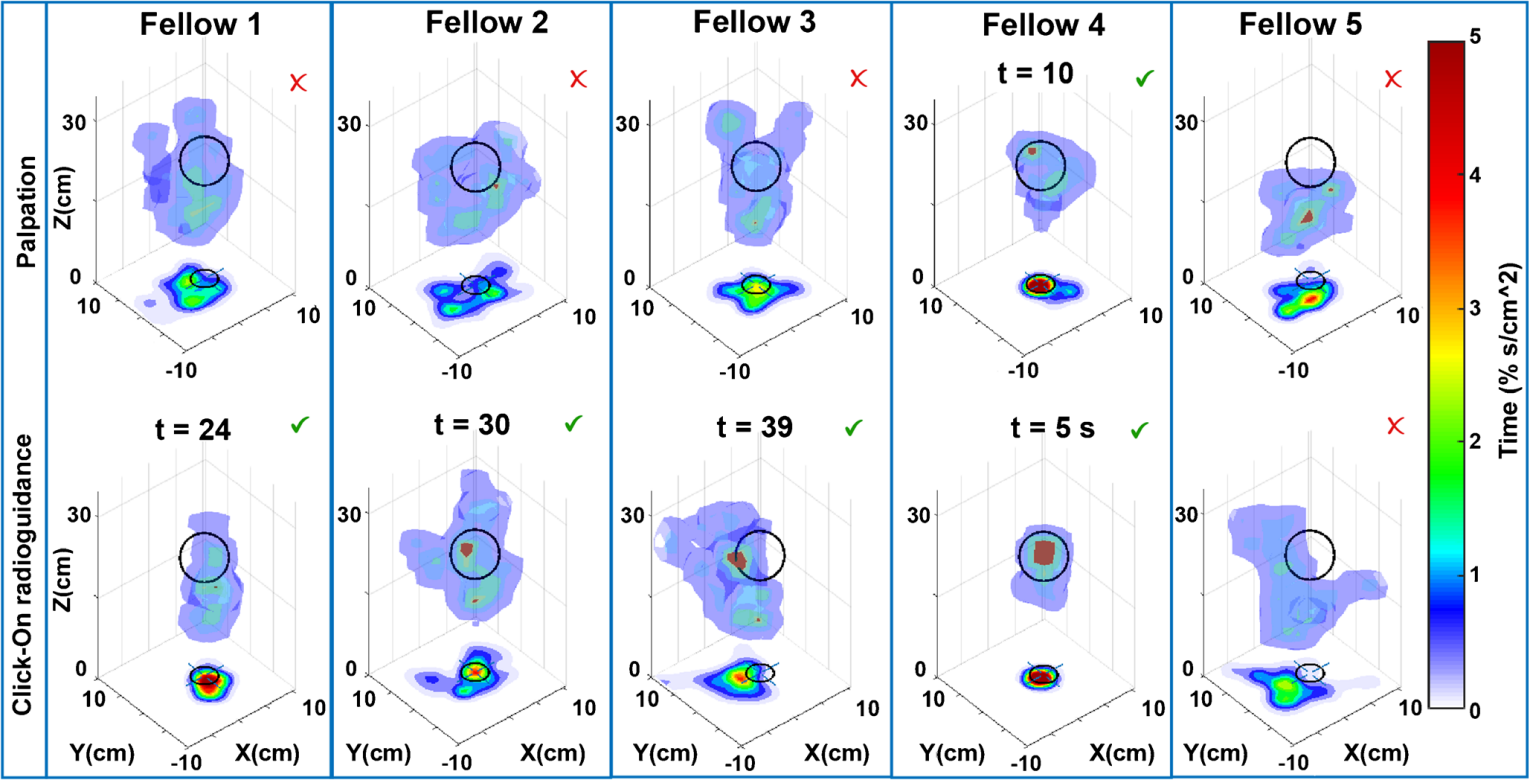
Track analysis of Click-On radioguidance in vivo during robot-assisted laparoscopic surgery. The 3D surface plots are based on the percentage of time spent in a specific location of the abdomen (%s/cm^3^) in each experiment. This is also converted into a color-coded 2D (%s/cm^2^; related to the color bar shown on the right) plots on the *XY* plane. Top row: the results of each fellow surgeon trial for finding the hidden target with palpation, bottom row: results of the same fellow surgeon with Click-On probe guidance (fellows 1 and 2 could not find the target without Click-On probe, they did find it using the probe guidance at 24 and 30 s, respectively; fellow 3 found the target with Click-On probe on the last seconds (*t* = 39 s); fellow 4 found it in both experiments very quickly; fellow 5 could not find it in both experiments)

**Fig. 5:**
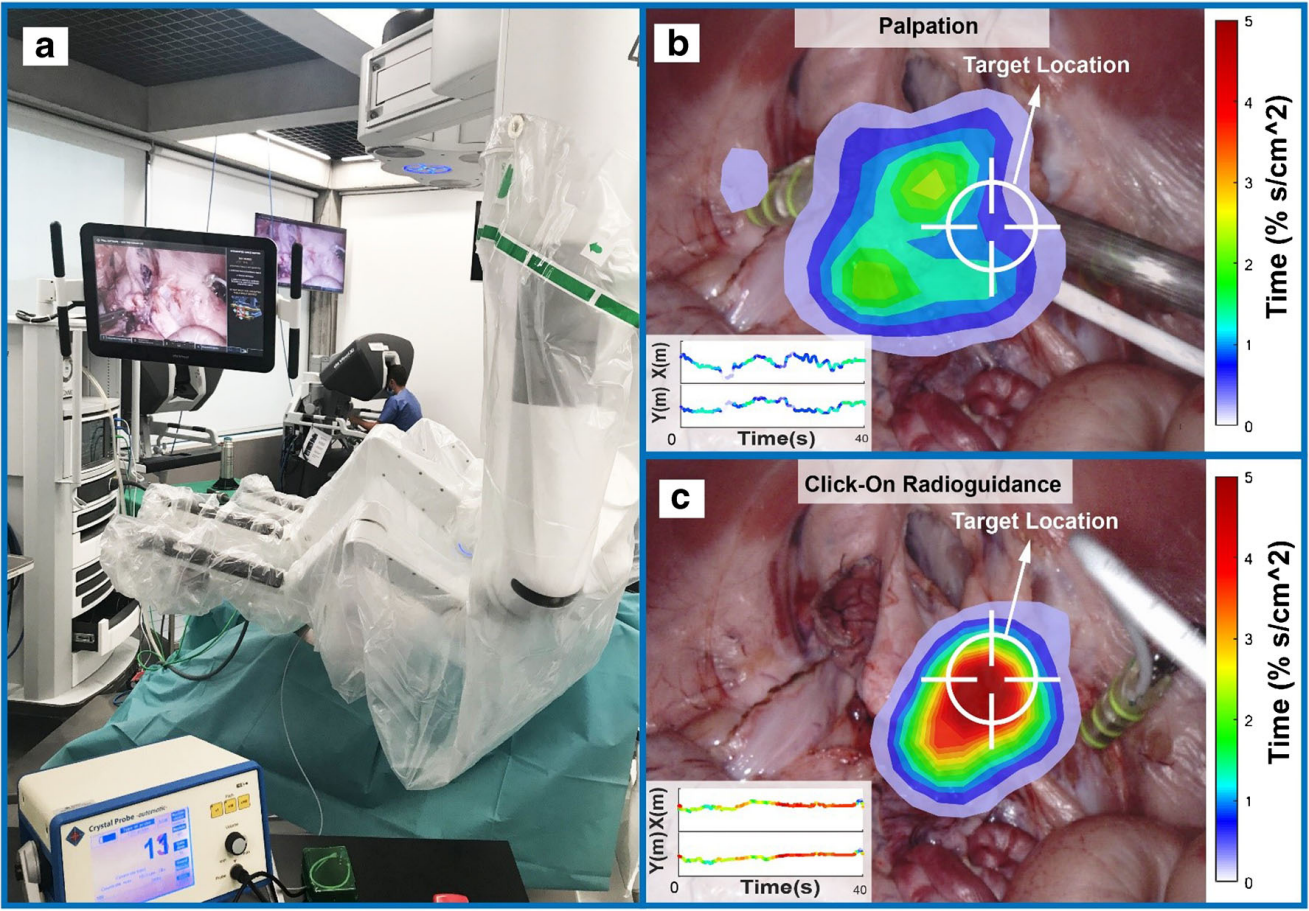
Augmented display of instrument positioning in the surgical field. **a** Overview of the operating room. **b**, **c** overlay of the density plots of *X* and *Y* components of the movement based on the time spent in each cm^2^ of the surgical view (example shown for one surgeon searching for the hidden target with palpation and Click-On probe radioguidance, respectively)

Using a blinded target identification exercise in robot-assisted porcine radioguided surgery, we were able to identify how Click-On radioguidance increases the surgeon’s ability to identify the target in space. Interestingly, the target discovery rate was 20% using visual inspection and increased up to 80% with radioguidance (Fig. 4), a rather stunning fourfold improvement in this critical decision-making process. Detection times under radioguidance varied between 5 and 39 s. The time spent in a specific location of the abdomen in 3D (Fig. 4) and 2D (Fig. 5) made it possible to compare individual surgeons’ performance. A valid decision with regard to the target location translated into a higher percentage of time spent at the location of the target. This increment in movement specificity suggests an enhancement in dexterity. No statistical differences could be found in path length, straightness index, angular dispersion, smoothness, curvature, instrument speed, and acceleration (see Table 1).

Through a basic machine learning algorithm, we were able to rank the surgical dexterity of the five surgeons with regard to accurate target definition. This scoring indicated that fellows 1, 4, and 2 performed better; and fellow 3 found the target on the last seconds. Conversely, fellow 5 failed to find the target even with guidance.

## Discussion

Image-guided surgery technologies intend to improve the surgeon’s ability to identify and optimally remove the tissue of interest [31]. In the current study, we were thus challenged by the question: How can we go above and beyond the recently CE-marked DROP-IN gamma probe concept that we have been exploring since 2014? [6]. We addressed this point by improving the ease-of-use via integration of the detector with the surgical instrument and by using video-based tool tracking to assess how the technology impacted on surgical decision-making and dexterity. Our findings suggest that engineering efforts focused on further integration between tethered gamma probes and laparoscopic surgical instruments positively impact target identification, procedural time, number of actions required to achieve a task, the path traveled by the surgical instruments, and movement patterns.

The new Click-On gamma probe concept proved to contain all the key benefits of the parental DROP-IN gamma probes (tethered, exploitation of the full rotational freedom of the robotic surgical instruments, autonomous probe positioning by the surgeon, and compatibility with a 12-mm trocar) complemented by a superior ease of use. During our dexterity experiment (superficial setting), this reflected through a 40% reduction in movements, which in turn translated to a significant three-time reduction in procedural time, traveled distance, and increase in straightness index (see Table 1). One may reason that the effect would be even more pronounced for identification of deep-seated lesions, where the procedure will demand further excision requiring a handling that benefits from the ProGrasp instrument being able to grasp the tissue. In fact, it is the preserved ability to hold on to tissue which sets the Click-On probe concept apart from its competition.

Our in vivo blinded exercise allowed us to demonstrate that the guidance provided by the Click-On gamma probe increased target identification from 20 to 80% when compared to using palpation only. This suggests that the information provided by the probe enhances the user’s ability to define a target location in space. Enhancement of this spatial ability impacts on dexterity [32–37], which was reflected in the more focused movements as a result of radioguidance with the Click-On (Figs. 4 and 5), a set-up that underlines the value of the guidance provided by gamma probes in general. Noteworthy to mention is that even without any prior radioguidance experience, the surgeons quickly adopted to use of the Click-On probe. The exploratory use of a machine-learning algorithm allowed us to effectively rank the performance of the individual surgeons in this group based on the movement features, a concept that in the future, using large datasets, could be used for personalized prediction of surgical skills.

Instrumental for our directionality-based video analysis has been the use of video-based instrument and probe tracking. To realize such tracking, we relied on a variation of the (fluorescently) colored marker–based tracking, which we previously reported for DROP-IN gamma probe navigation [24]. This helped us achieve a tracking accuracy in the (sub)-millimeter range (see above). We used a convenient asymmetric pattern of rings, but there are also literature reports using more complicated tracking patterns using, e.g., chessboard or circular dot patterns [25, 26, 38]. Alternatively, different groups have been promoting marker-less tracking of surgical instruments [39, 40]. Unfortunately, for the latter, there still are many challenges for the realization of automated instrumentation segmentation without the use of markers: robustness of segmentation, occlusion, lighting condition, and depth estimation (the reported tracking accuracy also is in the 0.25- to 5-mm range) [41–43]. It is, however, expected that for most video-based tracking methods (including marker and marker-less approaches), further improvements will follow using rapidly evolving machine-learning algorithms [39, 44]. Next to the analysis of movement patterns, instrument tracking obviously also creates the ability to apply gamma probes during “GPS-like” surgical navigation concepts [45]. For example, for the trackable DROP-IN design, we have been able to demonstrate how surgical navigation could help to integrate preoperative imaging (e.g., SPECT or PET scans) with intraoperative detection [24].

We are under the impression that analytical tools that help determine to what extent a new technology provides value in relation to the state-of-the-art can help catalyze clinical translation of new concepts and can help promote the adoption of new technologies in routine care. As with most initial reports of disruptive technologies, the initial findings presented here require further validation and final translation in the clinical setting. Such validation and translation should also investigate the impact of surgical decision-making on patient safety and surgical outcome. Obviously, the Click-On concept is by no means limited to the design of the ProGrasp instrument and can be adjusted to other instruments. It also seems reasonable to assume that the rather generic Click-On concept could in the future also help to increase the compatibility of alternative concepts such as beta detectors, ultrasound, confocal microscopy, and Raman spectrometry [46]. At the same time, tracking of the Click-On probe could also help rejuvenate concepts such as robotic-assisted laparoscopic SPECT [47].

## Conclusions

Designing a gamma probe that fully preserved the function of a wristed surgical ProGrasp instrument resulted in the tethered Click-On gamma probe. Using video-based track analysis, we provided preclinical evidence that the improved ease of use of this robotic-tailored tool improves dexterity and surgical decision-making.

## Data Availability

Available on reasonable request.
